# Development and Validation of a 1D Dynamic Model of an Injection Moulding Process and Design of a Model-Based Nozzle Pressure Controller

**DOI:** 10.3390/polym16101432

**Published:** 2024-05-18

**Authors:** Rasmus Aagaard Hertz, Ole Therkelsen, Søren Kristiansen, Jesper Kjærsgaard Christensen, Frederik Agervig Hansson, Lasse Schmidt

**Affiliations:** 1 R&D Moulding, LEGO System A/S, Åstvej 1, 7190 Billund, Denmark; 2 Materials Department, LEGO System A/S, Åstvej 1, 7190 Billund, Denmark; 3 Moulding Analytics Center, LEGO System A/S, Åstvej 1, 7190 Billund, Denmark; 4 Ngin A/S, Bautavej 1A, 8210 Aarhus, Denmark; 5 AAU Energy, Aalborg University, Pontoppidanstraede 111, 9220 Aalborg, Denmark

**Keywords:** injection moulding, process control, dynamic modelling, controller design, nozzle pressure control, experimental validation

## Abstract

A 1D model describing the dynamics of an injection moulding machine and the injection process is presented. The model describes an injection cylinder actuated by a dual-pump electro–hydraulic speed-variable drive and the filling, holding and cooling phases of the injection moulding process utilising amorphous polymers. The model is suggested as the foundation for the design of model-based pressure controllers of, e.g., the nozzle pressure. The focus is on using material, mould and machine properties to construct the model, making it possible to analyse and design the dynamic system prior to manufacturing hardware or conducting experiments. Both the presented model and the developed controller show good agreement with experimental results. The proposed method is general in nature and enables the design, analysis and evaluation of the machine, material and mould dynamics for controller design based solely on the physical properties of the system.

## 1. Introduction

Injection moulding is one of the main technologies used to produce plastic parts and enables the production of about one-third of plastic products. The production method is used in several sectors, such as the toy, food, medical and automotive sectors. The amount of plastic parts produced is growing due to their use in multiple different products, increasing the requirements for quality and tolerances [1,2,3]. Increased plastic consumption, together with a focus by the general public on sustainability in the sector, results in larger demand for both virgin and reused plastic feedstock. This requires manufactures to be able to produce using feedstock from multiple vendors or feedstock with increased variance between batches [4]. The success of injection moulded parts is dependent on the interplay of at least three different domains: the equipment, the process and the material. The increased variance either from vendor to vendor or batch to batch can possibly be solved in each category. The general aim of a control system is to manipulate the desired states to achieve a desired objective. A method to design a suitable controller is by understanding the system through a representative dynamic model [5].

Control of injection moulding machines has been a focus area since the 1980s, and the focus has been on the precision and repeatability of injection moulding machines [6]. The precision and repeatability of the machines should, if the boundary conditions are constant, result in the same part dimensions. In a production environment, maintaining constant boundary conditions is unlikely, as temperature, humidity and vendors of raw materials change over time [7].

To take the changing boundary conditions into account, the control structure is often separated into a cascaded control structure with three loops: machine control, process control and part control [8]. To increase the controllability of the injection moulding process, further research and development of the three loops are needed [9]. The goal of adding any controller to the injection moulding machine is to improve the quality or quantity of the product. The possibility of measuring directly on the output can be limited; however, the general reasoning is that it is an improvement to go from machine control to process control and from process control to part control in order to decrease the variance of each produced part [8,10]. Multiple dynamic models for control design of injection moulding processes have previously been proposed in the literature. The genesis of a system model can be divided into two main categories: modelling and identification [5]. Models based on identification require a priori knowledge of the process, as model development is based on experiments. This comes at the cost of a loss of generalizability to other configurations and the need to have a setup to perform the experiments on [11]. Modelling is based on breaking a system up into submodels for which the properties are understood from previous knowledge, e.g., laws of nature or generic laboratory measurements. This, however, can be done without having a setup to conduct experiments, making it possible to generalise to comparable systems by changing relevant subsystems and parameters [5].

As the mould is dependent on the part produced, it is desired to be able to design the controller when the mould is designed and not await experiments with the mould in order to create a model. The focus when creating a dynamic model is to base it on material properties, mould geometries and machine dynamics. This enables users to test the influence of changing, e.g., material from simulation only, based on standard material test data such as viscosity, pvT and specific heat. This paper focus on process control, and the development of a 1D dynamic model of the moulding process is presented inspired by the work of [12,13,14,15]. The model is developed with the purpose of controller design, meaning the dynamic behaviour is of greater importance than the absolute precision. This is contrary to the focus area and achievements of, e.g., commercial software for simulation of the moulding process and the produced elements. The presented model contains the dynamics of both the novel electro–hydraulic dual-pump-driven injection moulding machine and the filling, holding and cooling phases of the injection moulding process. The design of a simple, model-based PI nozzle pressure controller based on the model is presented, followed by a comparison of both the model and controller performance with experimental data.

## 2. Previous Work

Modelling and control of the moulding process is an area that has been of interest for several decades; more-general literature reviews can be found in [8,16,17]. Early work with simulation of the injection moulding process started in the 1950s, when Spencer and Gilmore [18] investigated the filling phase. McKelvey [19] and Pearson [20] used a fundamental transport theorem to describe the process. A numerical solution was reported by Harry and Parrott [21], Kamal and Kenig [22], Kamal and Kenig [23]. Work with simulations has continued to present day [24,25,26,27,28]. Increases in computational power resulted in commercial programs, where modelling often relies on computational fluid dynamics (CFD) [29]. These programs analyse the behaviour of the dynamic system through a meshing, which introduces a large set of equations. The computational demands of these models, coupled with their emphasis on spatial distribution rather than dynamic behaviour, render these methods impractical for control design purposes [17].

Historically, control design has been based on empirical models, with the limit being that the model only describes the system dynamics at the operating points for which experiments have been performed [30,31,32,33]. Shankar and Paul [12] recognised that a general lumped parameter model of the machine, polymer and mould creates a good foundation for control design, machine component selection and process control without having to build a test system and conduct experiments. The model is a 1D lumped parameter model consisting of ordinary differential equations and algebraic equations. The flow is calculated based on equivalent resistances. The model is seen as two volumes: the antechamber and the cavity. The approach with two cavities is widely used in, e.g., [34,35,36]. Rafizadeh et al. [37,38] describes the filling phase and holding phase with two models, wherein the pressure drop along the flowpath is described by the power law model for non-Newtonian fluids. Chiu et al. [13] developed a similar model to that of Shankar and Paul [12] with the goal of investigating the relationship between the melt flow rate and the peak cavity pressure for an in-line quality controller. Wei et al. [39] extended the model by also considering the dynamics of a nonlinear servo–pump system. The model proposed by Woll and Cooper [15] focuses on a semi-crystalline material, whereby it takes into account the heat generated in the crystallization phase. It further introduces the material parameters through a one-domain Tait and cross-viscosity model, making it possible to generate cavity pressure patterns based on machine settings. The focus of the model is to build an in-line part-quality monitoring and control scheme. Lin and Cheng [40] used a similar approach as that of Chiu et al. [13] for the generation of virtual pressure sensors. Recently, Froehlich et al. [17] suggested using a phenomenological process model together with a detailed physics-based model of the servo-drive. The phenomenological process model, however, is based on resistances extracted from experiments, which is an empirical model of the moulding process.

Nozzle pressure control can be an advantage over hydraulic pressure control as it bypasses some of the errors, such as, e.g., uncertainty in the leakage over the check ring [41]. This work is concerned with both model development and model-based control design. The literature describing nozzle pressure control started in the 1980s when Fara et al. [31] developed a PID nozzle pressure controller based on an empirical model of the moulding process without a mould, showing improved response compared to open loop tests. Kamal et al. [32] designed both PID and Dahlin controllers based on a stochastic process model that included a mould. Both controllers show tracking capability. Yang and Gao [42] designed an adaptive nozzle pressure controller that showed improved results compared to a PID controller for several moulds based on an ARX model.

The existing literature shows a gap when it comes to a general approach to design a representative dynamic model without experimental validation and the adoption of a model-based strategy for nozzle pressure control design. These aspects are necessary if it is desired to design nozzle pressure controllers before the mould is manufactured and the full setup is created. The success of a control system is strongly linked to the ability to get reliable and time-sensitive measurement of key states in the process; reviews of current sensing possibilities are found in [2,43].

## 3. Model Structure

**Figure 1:**
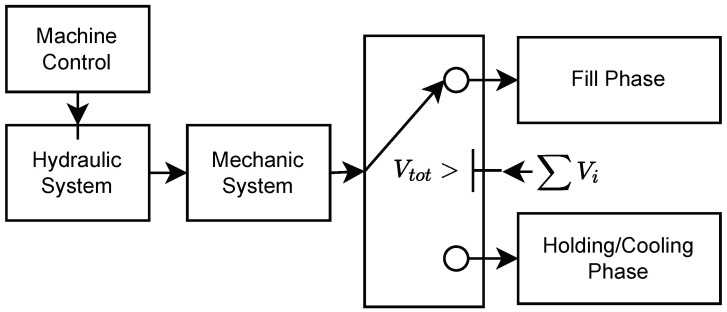
Structure of the presented model.

The proposed method described in this work is used to model a retrofitted electro–hydraulic speed-variable industrial injection moulding machine. The material utilised is an amorphous acrylonitrile butadiene styrene (ABS). The mould is a single-cavity cold-runner mould that produces a rectangular element. The model is assembled from several submodels. An overview of the model structure can be seen in Figure 1.

**Figure 2:**
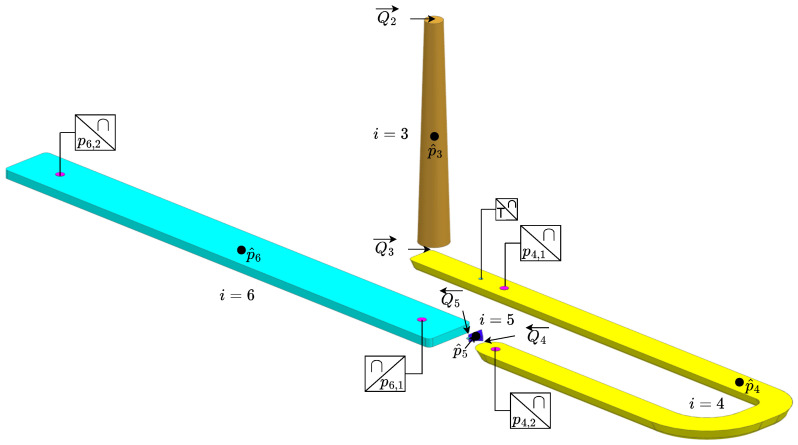
Flamebar element used for modelling. The volumes are numbered with the mathematical operator “*i*”. The modelled pressure is at the centre of each section, denoted by ${\hat{p}}_{i}$. The physical pressure sensors in the mould are marked as $p_{i,j}$. The arrows above the flows indicate the positive direction.

**Figure 3:**
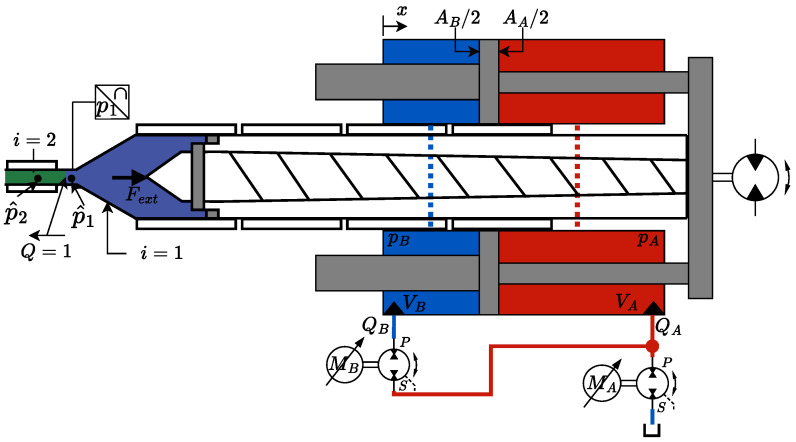
Simplified schematic of the hydraulic and mechanical subsystem.

The injection moulding machine is first considered, including the control of the machine. The injection moulding process is divided into two parts: a fill phase and a combined holding and cooling phase. For both models, the total volume of the mould is split up into six subvolumes, counted by the mathematical operator “*i*”. The subvolumes are the nozzle (i=1), extender (i=2), sprue (i=3), runner (i=4), gate (i=5) and cavity (i=6). An overview of the volumes can be seen in Figure 2 and Figure 3, where each volume is separated by colour. For each section, the pressure is modelled for the centre of the section, denoted ${\hat{p}}_{i}$, whereas the experimental data are measured at the spots marked by $p_{i,j}$. The arrows above the flow indicate if the flow is entering or leaving the volume, e.g., $Q_{4}$ is going from the runner (i=4) to the gate (i=5). The symbols used in the equations describing the model can be found in the nomenclature list.

## 4. Hydraulic System

The injection moulding machine is a retrofitted hydraulic industrial injection moulding machine. The injection unit is driven by a electro–hydraulic variable-speed drive. The dual-pump concept is described in further detail in [44,45,46]. A simplified schematic of the hydraulic and mechanical subsystem is seen in Figure 3. The chamber dynamics are given in Equations (1) and (2).

(1) $$\begin{array}{cc} {\dot{p}}_{A} & =\frac{\beta_{A}}{V_{A}}\left(Q_{A}-Q_{B}-Q_{Lc}+A_{A}\dot{x}\right) \end{array}$$

(2) $$\begin{array}{cc} {\dot{p}}_{B} & =\frac{\beta_{B}}{V_{B}}\left(Q_{B}+Q_{Lc}-A_{B}\dot{x}\right) \end{array}$$

The movement of the ram can be described by Newton’s second law of motion as Equation (3):

(3) $$\begin{array}{c} \ddot{x}=\frac{1}{m}\left(-p_{A}A_{A}+p_{B}A_{B}-F_{C}sgn\left(\dot{x}\right)-B_{v}\dot{x}+F_{ext}\right) \end{array}$$

The external force is generated from the molten polymer in the nozzle as $F={\hat{p}}_{1}A_{c,1}$, where ${\hat{p}}_{1}$ is derived from Equation (9). The modelling and control strategy of the hydraulic subsystem is based on physically desired control principles according to the methodology described in [47]. This is contrary to sophisticated control algorithms like [48,49,50]. The objective is to control the system in such a manner that the pressure dynamics of two virtual states behave as a first-order system. The two virtual states are the load pressure and the level pressure, making it possible to control both the pressure level in the system and the force on the piston. The load pressure and level pressure are shown in Equation (4).

(4) $$\begin{array}{c} p_{L}=p_{A}-\alpha p_{B},\;\;\;\;\;p_{H}=p_{A}+p_{B} \end{array}$$

The desired pressure dynamics are stated in Equation (5):

(5) $$\begin{array}{c} {\dot{p}}_{L}={\dot{p}}_{A}-\alpha {\dot{p}}_{B},\;\;\;\;\;{\dot{p}}_{H}={\dot{p}}_{A}+{\dot{p}}_{B},\;\;\;\;\;\alpha =\frac{A_{B}}{A_{A}} \end{array}$$

From this, the reference to each motor can be calculated; the motor reference for each motor is shown in Equations (6)–(8):

(6) $$\begin{array}{cc} \omega_{A} & =\psi_{1}\left(-p_{L}^{*}+p_{L}\right)+\psi_{2}\left(p_{H}^{*}-p_{H}\right)-\frac{\left(A_{A}-A_{B}\right)}{D_{A}}\tilde{\dot{x}} \end{array}$$

(7) $$\begin{array}{cc} \omega_{B} & =\psi_{3}\left(p_{L}^{*}-p_{L}\right)+\psi_{3}\left(p_{H}^{*}-p_{H}\right)+\frac{A_{B}}{D_{B}}\tilde{\dot{x}} \end{array}$$

where

(8) $$\begin{array}{cc} \psi_{1} & =\frac{\omega_{L}\left(V_{A}-V_{B}\right)A_{A}}{\tilde{\beta }\left(A_{A}+A_{B}\right)D_{A}},\;\;\;\;\;\psi_{2}=\frac{\omega_{H}\left(A_{A}V_{B}+A_{B}V_{A}\right)}{\tilde{\beta }\left(A_{A}+A_{B}\right)}\\ \psi_{3} & =\frac{V_{B}A_{A}}{\tilde{\beta }\left(A_{A}+A_{B}\right)D_{B}} \end{array}$$

where $\tilde{\dot{x}}$ is the estimated velocity: in the case, for which the motion controller is active, $\tilde{\dot{x}}$ is the velocity reference. The estimated velocity equals zero when only the load pressure control is active, as there is no velocity trajectory. $\tilde{\beta }$ is the estimated bulk modulus.

The filling phase of the moulding process is typically velocity controlled, whereas the holding phase is pressure controlled. A proportional-integral (PI) velocity controller is derived, together with a position controller. The trajectory is designed to be smooth and differentiable, with a path that begins with a zero initial condition for the position and its derivatives up to the sixth derivative, commonly referred to as the piston’s “crackle” [51,52]. The switchover to the holding pressure is based on a low-pass filter, ensuring a smooth transition; further details are given in Hertz et al. [45].

## 5. Filling Phase

In the filling phase, the injection ram moves forward and the melt fills the mould. Until the mould is full, it is assumed that the melt pressure at the flow front is atmospheric pressure. This assumption is valid if the mould is well vented. It is further assumed that the pressure drop along the nozzle is negligible. The flow out of the nozzle depends on the mould and the force from the hydraulic system. The continuity equation is used to calculate the pressure at the centre of the nozzle according to Equation (9) and Figure 3.

(9) $$\begin{array}{c} {\dot{\hat{p}}}_{1}=\frac{\beta_{1}}{V_{1}}\left(A_{c,1}\dot{x}-Q_{1}\right) \end{array}$$

where $\beta_{1}$ is the bulk modulus of the melt, $\dot{x}$ is the ram velocity, $V_{1}$ is the volume of the melt, and $Q_{1}$ is the flow out of the nozzle. The volume of the nozzle changes according to the cylinder position, as shown in Equation (10):

(10) $$\begin{array}{c} V_{1}=V_{1,I}-A_{c,1}x \end{array}$$

where $V_{1,I}$ is the initial volume of the nozzle, and $A_{c,1}$ is the cross-sectional area of the nozzle. Adiabatic compressibility can, according to Praher et al. [53], be described by Equation (11).

(11) $$\begin{array}{c} \kappa_{i}=\frac{1}{\nu (T_{i},{\hat{p}}_{i})}\left({\left(\frac{\partial \nu (T_{i},{\hat{p}}_{i})}{\partial {\hat{p}}_{i}}\right)}_{T_{i}=T_{0}}+\left(\frac{T_{i}}{c_{p}}\right){\left(\frac{\partial \nu (T_{i},{\hat{p}}_{i})}{\partial T}\right)}_{p=p_{0}}^{2}\right) \end{array}$$

The specific volume can be retrieved from the equation of state, which is described by pvT diagrams. A pvT diagram relates pressure, specific volume and temperature and can be represented by empirical equations. Multiple formulations of the equation are presented in the literature, e.g., the Schmidt model and the one-domain Tait and two-domain Tait equations [54]. With the two-domain Tait equation, it is possible to describe the pvT relationship for a wide variety of plastics in the solid and melt states [55,56]. For amorphous materials, the two-domain Tait model has proven successful, as it, e.g., describes the transition at the glass transition temperature. The two-domain Tait equation is given in Equation (12).

(12) $$\nu \left(T_{i},{\hat{p}}_{i}\right)=\nu_{0}\left(T_{i}\right)\left(1-C\ln\left(1+\frac{{\hat{p}}_{i}}{B(T_{i})}\right)\right)+\nu_{1}\left(T_{i},{\hat{p}}_{i}\right)$$

The temperature of the polymer in the filling phase is assumed constant, as it has been found that the cooling of the polymer is counteracted by the generated shear heat in the filling phase [12]. When the polymer is injected into the mould, it is in the molten state, meaning only the equations related to the liquid state are used in the filling phase to calculate the bulk modulus shown in Equation (14). However equations for both states are shown for later reference in Equations (13) and (14).

(13) $$\begin{array}{cc} \mathrm{For}\;T_{i} & <b_{5}+b_{6}{\hat{p}}_{i}\\ \; & \nu_{0}\left(T_{i}\right)=b_{1s}+b_{2s}\left(T_{i}-b_{5}\right)\\ \; & B\left(T_{i}\right)=b_{3s}e^{-b_{4s}\left(T_{i}-b_{5}\right)}\\ \; & \nu_{1}\left(T_{i},p_{i}\right)=b_{7}e^{b_{8}\left(T_{i}-b_{5}\right)-b_{9}\hat{p}} \end{array}$$

(14) $$\begin{array}{cc} \mathrm{For}\;T_{i} & \geq b_{5}+b_{6}{\hat{p}}_{i}\\ \; & \nu_{0}\left(T_{i}\right)=b_{1m}+b_{2m}\left(T_{i}-b_{5}\right)\\ \; & B\left(T_{i}\right)=b_{3m}e^{-b_{4m}\left(T_{i}-b_{5}\right)}\\ \; & \nu_{1}\left(T_{i},p_{i}\right)=0 \end{array}$$

The inverse of the adiabatic compressibility is the bulk modulus according to [57], and it is shown in Equation (15).

(15) $$\begin{array}{c} \beta_{i}=\frac{1}{\kappa_{i}} \end{array}$$

The two parameters $\frac{\partial \nu }{\partial \hat{p}}$ and $\frac{\partial \nu }{\partial T}$ can be calculated based on Equations (12) and (14). The flow out of the nozzle $Q_{1}$ is according to Woll and Cooper [15], described by the flow gradient assuming laminar flow and an incompressible fluid by Equation (16).

(16) $$\begin{array}{c} {\dot{Q}}_{1}=\frac{{\hat{p}}_{1}-p_{ff}-\sum_{i=1}^{6}\frac{F_{s,i}}{A_{c,i}}}{\rho_{avg}\sum_{i=1}^{6}\frac{H_{L,i}}{A_{c,i}}} \end{array}$$

where ${\hat{p}}_{1}$ is the nozzle pressure, $p_{ff}$ is the pressure of the flow front, $F_{s,i}$ is the resistive shear force of each section, $H_{L,i}$ is the hydraulic length of each section, $A_{c,i}$ is the cross-sectional area of each section, and $\rho_{avg}$ is the average melt density in the nozzle and mould. The melt density and average melt density are calculated from the Tait equation and are given by Equations (17) and (18), respectively.

(17) $$\begin{array}{cc} \rho_{i} & =\nu {\left({\hat{p}}_{i},T_{0}\right)}^{-1} \end{array}$$

(18) $$\begin{array}{cc} \rho_{avg} & =\nu {\left(\frac{{\hat{p}}_{1}-p_{ff}}{2},T_{0}\right)}^{-1} \end{array}$$

The resistive shear force represents the resistance to flow and is different for each section. The resistive shear force is dependent on the surface area of section $A_{s,i}$ and the shear stress of section $\tau_{rz,i}$, as given in Equation (19).

(19) $$\begin{array}{c} F_{s,i}=A_{s,i}\tau_{rz,i} \end{array}$$

The shear stress of the section is given by Equation (20):

(20) $$\begin{array}{c} \tau_{rz,i}=\eta_{i}{\dot{\gamma }}_{i} \end{array}$$

where $\eta_{i}$ is the viscosity of the i-th section, and ${\dot{\gamma }}_{i}$ is the shear rate. The exact shear rate ${\dot{\gamma }}_{i}$ is dependent on the shape of the channel. However, it is approximated based on Equation (21).

(21) $$\begin{array}{c} {\dot{\gamma }}_{i}=\frac{32\cdot abs(Q_{i})}{\pi D_{H,i}^{3}} \end{array}$$

The viscosity is calculated from the cross-WLF viscosity model given in Equation (22).

(22) $$\begin{array}{c} \eta =\frac{\eta_{0}}{1+{\left(\frac{\eta_{0}\dot{\gamma }}{\tau^{*}}\right)}^{1-n}},\;\;\;\;\eta_{0}=Be^{\frac{T_{b}}{T_{i}}} \end{array}$$

The flow and pressure of the fill phase are described; however, they are based on the hydraulic dimensions, which are considered next.

## 6. Hydraulic Dimensions

The mould is separated into multiple volumes as the geometry changes in the mould, as shown in Equation (16). The hydraulic flow length $H_{L,i}$, surface area $A_{s,i}$ and cross-sectional area are dependent on the melt-front position. Volumetric filling of the mould is dependent on the flow into the mould and is given in Equation (23).

(23) $$\begin{array}{c} \dot{V}=Q_{1} \end{array}$$

The initial volume is given as $V_{2,t\;=\;0}=V_{2}-A_{c,1}x_{dec}$; this accounts for the decompression after plasticising. The hydraulic dimensions of each segment in the mould are shown in Table 1. The hydraulic diameter of each section is used and is calculated for the different geometries, as shown in Equation (24).

(24) $$\begin{array}{cc} D_{H,i} & =\frac{4A_{c,i}H_{T,i}}{A_{s,i}} \end{array}$$

For i=2,4,5,6 and if the melt has reached that segment, the instantaneous flow length and volume are calculated as scaling, according to Equation (25):

(25) $$\begin{array}{cc} H_{L,i} & =H_{t,i}\frac{V_{i}}{V_{t,i}}\;\;\;,\;\;\;V_{i}=V-\sum_{j=1}^{j=i-1}V_{t,j}\\ A_{s,i} & =A_{s,i}\frac{H_{L,i}}{H_{t,i}} \end{array}$$

In the holding phase, when volumetric filling of the mould is complete, the molten volume of the polymer is calculated according to Equation (26).

(26) $$\begin{array}{c} V_{H,i}=\frac{\pi \cdot D_{H,i}^{2}}{4} \end{array}$$

Remark: This will create a discontinuity as $V_{i}\neq V_{H,i}$, as the hydraulic radius is an approximation from the given geometry. This, however, does not influence the model as it happens as the model switches from the filling phase to the holding phase.

The sprue (i=3) is a special geometry that is considered differently. For further information about the calculation of volumes, areas and hydraulic diameters of the sprue, see Appendix A.

### Initial Conditions for the Packing Phase

As the packing phase considers the pressure dynamics of each section separately, it is necessary to calculate the initial pressure of each section. First, consider the pressure drop calculated from the Hagen–Poiselle pressure drop for each section, as shown in Equation (27).

(27) $$\begin{array}{c} \Delta p_{i}=\frac{128\eta_{i}Q_{1}H_{L,i}}{\pi D_{H,i}^{4}} \end{array}$$

As the initial condition for the holding pressure phase should describe the pressure at the centre of each section, and Hagen–Poiselle describes the total pressure drop in each section, an additional step is necessary. The pressure at the centre of each section i=2…6 is calculated according to Equation (28).

(28) $$\begin{array}{c} p_{i}=p_{i-1}-\left(\frac{\Delta p_{i-1}}{2}+\frac{\Delta p_{i}}{2}\right)\left(\frac{p_{1}}{\sum_{i=1}^{6}\Delta p_{i}}\right) \end{array}$$

For i=1, which is the pressure at the centre of the nozzle, the pressure is given in Equation (9). This concludes the model of the filling phase.

## 7. Packing Phase

The switchover to the packing phase occurs as the volume of injected material equals the total volume of the mould. In the packing phase, pressure is kept on the nozzle to compensate for the shrinkage in the mould. It is not possible to assume that the pressure at the flow front is atmospheric, and the melt is incompressible, meaning flow $Q_{1}\neq Q_{i}$ anymore. Instead, the pressure dynamic is set up for each section i=2⋯6 of the mould. The nozzle and extender are heated, meaning the polymer in the extender will stay at the melt temperature. The pressure dynamics are similar to those of the nozzle and are given in Equation (29)

(29) $$\begin{array}{c} {\dot{\hat{p}}}_{2}=\frac{\beta_{2}\left(\hat{p},T\right)}{V_{2}}\left(Q_{1}-Q_{2}\right) \end{array}$$

whereas for section i=3⋯6, the material temperature changes. The density of the polymer material changes with temperature and pressure; however, that is not captured directly in the continuity equation. To account for this effect, a leakage term is introduced as $\frac{1}{\nu_{p}}\dot{\nu }m$ given in Equation (30).

(30) $$\begin{array}{c} {\dot{\hat{p}}}_{i}=\frac{\beta_{i}\left(\hat{p},T\right)}{V_{i}}\left(Q_{i-1}-Q_{i}+\frac{1}{\nu_{p}}\dot{\nu }m_{i}\right) \end{array}$$

where $Q_{i-1}$ is the flow into the section, and $Q_{i}$ is the flow out of the section. For i=6, the flow out is $Q_{6}=0$. The term $\dot{\nu }$ is the change in specific volume and is a function of both pressure and temperature; however, as the pressure changes slowly in the cooling phase, it is assumed that $\frac{d\hat{p}}{dt}=0$. The flow of each segment i=2⋯6 is updated according to Equation (31).

(31) $$\begin{array}{c} {\dot{Q}}_{i-1}=\frac{{\hat{p}}_{i-1}-{\hat{p}}_{i}-\frac{1}{2}\sum_{i-1}^{i}\left(\frac{F_{s,i}}{A_{c,i}}\right)}{\frac{\rho_{i-1}+\rho_{i}}{2}\sum_{i-1}^{i}\left(\frac{H_{L,i}}{A_{c,i}}\right)} \end{array}$$

Having determined all pressures and flows, it is necessary to understand the cooling of the polymer, as this will restrict the flow and thereby limit the pressure as the polymer solidifies.

### Cooling

The cooling of the channel is also considered in the 1D domain, which is a rather crude simplification. The simplification is based on several key assumptions, including the following.

- The mould has constant a temperature independent of position;
- The heat flux is perpendicular and is equal in all directions at any given position;
- The heat from shear and inflow of hot material in the packing phase is not considered.

It is furthermore assumed that the shear heat is equal to cooling in the filling stage of the mould, meaning cooling starts as soon as the mould is full at the same instance the holding phase begins. This is shown to be a good assumption by Shankar and Paul [12].

The temperature of each section is considered individually as a one-dimensional transient heat conduction problem of a cylinder. Assuming L>>d, no heat generation, thermal symmetry about the midplane, uniform initial temperature, and a constant convection coefficient it is according to Hopmann et al. [58] possible to calculate the average temperature of each section from Equation (32).

(32) $$\begin{array}{c} {\bar{T}}_{i}=0.692\left(T_{p}-T_{W}\right)e^{\frac{-23.14\cdot t\cdot \alpha_{p}}{D_{H,i}^{2}}}+T_{W}\;,\;\;\;\alpha_{p}=\frac{k_{p}}{\rho_{i}c_{p}} \end{array}$$

As the gate (i=5) does not fulfil the property L>>r, the thermal heat conductivity is divided by two to take into account end effects. The interface temperature $T_{w}$ between the steel and the polymer is according to Wang et al. [54] given by Equation (33).

(33) $$\begin{array}{c} T_{W}=\frac{b_{s}T_{s}+b_{p}\cdot {\bar{T}}_{I}}{b_{s}+b_{p}} \end{array}$$

where $b_{p}$ and $b_{s}$ are the thermal effusivities of the polymer and the mould material, respectively, $\bar{T}$ is the average melt temperature, and $T_{s}$ is the the cooling water temperature. The initial average temperature needs to be equal to the temperature in the injection phase to ensure a continuous temperature profile in both the injection and holding phases. The peak temperature $T_{p}$ can now be calculated, as the initial average temperature is known. As the material cools, the hydraulic diameter tends to zero. The term ψ describes the frozen layer where the flow of polymer is not possible and is given in Equation (34).

(34) $$\begin{array}{c} \psi_{i}=\frac{\bar{T}-T_{g}}{{\bar{T}}_{I}-T_{g}}-1 \end{array}$$

where $T_{g}$ is the glass transition temperature, and ${\bar{T}}_{I}$ is the initial average temperature when the packing phase starts. The hydraulic diameter is updated according to Equation (35).

(35) $$\begin{array}{c} D_{\psi ,i}=D_{H,i}\sqrt{1-\psi_{i}} \end{array}$$

The surface area and cross-sectional area are updated according to Equation (36).

(36) $$\begin{array}{c} A_{s,i}=D_{\psi ,i}\pi H_{L,i},\;\;\;\;\;\;A_{c,i}=\frac{D_{\psi ,i}^{2}\pi }{4} \end{array}$$

All governing equations are presented. The last thing to consider is the singularity in the solution when the gate is completely frozen, as per Equation (37).

(37) $$\begin{array}{c} \lim_{\psi_{i}\to 1}D_{\psi ,i}\left(\psi_{i}\right)=0 \end{array}$$

To avoid this, it is defined that the flow $Q_{5}=0$ for $\psi_{5}<0.15$, resulting in the only change in pressure being attributed to shrinkage after gate closure. The design of the controller based on the model is discussed first before the design of the experimental validation of the proposed model is discussed.

## 8. Control

The nonlinear model described in Section 4, Section 5, Section 6 and Section 7 is both nonlinear and time-varying. This needs to be considered when designing controllers to ensure stability at all times. The benefit of linear controllers such as PI controllers is that they are well understood, the response is robust, and it is possible to analyse and tune them with simple guidelines. This is contrary to, e.g., fuzzy logic controllers, where tuning is based on trial and error [59].

### 8.1. Model Linearisation

In the analysis of linear controllers, a linear plant serves as a fundamental prerequisite. The process of linearizing the nonlinear model is outlined below. The described nonlinear model is linearised utilising a Taylor series. While the model consists of a fill and pack phase, they are considered individually.

#### 8.1.1. Fill Phase

The governing equations describing the fill phase are given in Equations (9)–(22). The state vector is defined as $x_{Fill}={\left[\begin{array}{cccc} x & \dot{x} & {\hat{p}}_{1} & Q_{1} \end{array}\right]}^{T}$. It is further assumed that the bulk modulus of the melt can be approximated as $\beta_{1}=K\cdot {\hat{p}}_{1}$. The Taylor expansion for the nozzle pressure gradient and flow dynamics are given in Equation (38).

(38) $$\begin{array}{cc} \Delta {\dot{\hat{p}}}_{1L}= & \frac{\partial {\dot{\hat{p}}}_{1}}{\partial x}{|}_{x_{Fill,0}}\Delta x+\frac{\partial {\dot{\hat{p}}}_{1}}{\partial \dot{x}}{|}_{x_{Fill,0}}\Delta \dot{x}+\frac{\partial {\dot{\hat{p}}}_{1}}{\partial p_{1}}{|}_{x_{Fill,0}}\Delta {\hat{p}}_{1}+\frac{\partial {\dot{\hat{p}}}_{1}}{\partial Q_{1}}{|}_{x_{Fill,0}}\Delta Q_{1}+\frac{\partial {\dot{\hat{p}}}_{1}}{\partial p_{L}}{|}_{x_{Fill,0}}\Delta p_{L}\\ \Delta {\dot{Q}}_{1L}= & \frac{\partial {\dot{Q}}_{1}}{\partial x}{|}_{x_{Fill,0}}\Delta x+\frac{\partial {\dot{Q}}_{1}}{\partial \dot{x}}{|}_{x_{Fill,0}}\Delta \dot{x}+\frac{\partial {\dot{Q}}_{1}}{\partial p_{A}}{|}_{x_{Fill,0}}\Delta p_{1}+\frac{\partial {\dot{\hat{p}}}_{1}}{\partial p_{B}}{|}_{x_{Fill,0}}\Delta Q_{1}+\frac{\partial {\dot{Q}}_{1}}{\partial p_{L}}{|}_{x_{Fill,0}}\Delta p_{L} \end{array}$$

where $x_{Fill,0}={\left[\begin{array}{ccccc} x_{0} & {\dot{x}}_{0} & {\hat{p}}_{10} & Q_{1O} & p_{L0} \end{array}\right]}^{T}$ is the equilibrium point. The piston position will vary together with the volume of polymer in the mould and barrel. The term $x_{Fill,0}$ is determined based on a pole sweep at five distinct points. The five points are when each section from the nozzle to the cavity is full. The position with lowest damping is selected, which is when the mould is full. The holding pressure set point is used as the linearisation point of the load pressure $p_{L0}=p_{Hold}$ bar. From this, it is possible to calculate the equilibrium point from $\ddot{x}=0$, ${\dot{\hat{p}}}_{1}=0$ and ${\dot{Q}}_{1}=0$ given in Equation (39).

(39) $$\begin{array}{cc} x_{0}= & x_{s}-x_{dec},\;\;\;{\dot{x}}_{0}=0,\;\;\;p_{L0}=p_{Hold}^{*},\;\;\;{\hat{p}}_{10}=\frac{A_{A}p_{L0}}{A_{1}},\;\;\;Q_{10}=0 \end{array}$$

The linearised barrel volume is given in Equation (40).

(40) $$\begin{array}{cc} V_{1,0} & =V_{1,I}+A_{1}x_{0} \end{array}$$

The state space representation of the linearised model of the fill phase is given in Equation (41).

(41) $$\begin{array}{cc} \; & {\dot{x}}_{Fill}=A_{Fill}x_{Fill}+B_{Fill}u_{Fill},\;\;\;y_{Fill}=C_{Fill}x_{Fill},\;\;\;x_{Fill}={\left[\begin{array}{cccc} x & \dot{x} & {\hat{p}}_{1} & Q_{1} \end{array}\right]}^{T}\\ \; & u_{Fill}={\left[\begin{array}{c} p_{L} \end{array}\right]}^{T},\;\;\;A_{Fill}=\left[\begin{array}{cccc} \;0 & 1 & 0 & 0\\ \;0 & -\frac{B_{v}}{m} & -\frac{A_{c,1}}{m} & \frac{A_{B}}{m}\\ \;0 & -\frac{\beta_{1,0}A_{1}}{V_{1,0}} & 0 & -\frac{\beta_{1,0}}{V_{1,0}}\\ \;0 & 0 & \frac{A_{c,1}}{\rho_{avg,0}H_{L,1}} & -\frac{32A_{s,1}\eta_{0}}{\pi D_{H,i}\rho_{avg,0}H_{L,1}} \end{array}\right]\\ \; & B_{Fill}{\left[\begin{array}{cccc} \;0 & \frac{-A_{A}}{m} & 0 & 0 \end{array}\right]}^{T},\;\;\;C_{Fill}\left[\begin{array}{cccc} 0 & 0 & 1 & 0 \end{array}\right] \end{array}$$

From the state space representation of the system, the transfer function between the hydraulic load pressure and the nozzle pressure can be derived as shown in Equation (42).

(42) $$\begin{array}{c} G_{Fill}=C_{Fill}{\left(sI_{Fill}-A_{Fill}\right)}^{-1}B_{Fill} \end{array}$$

This finalises the linear model of the fill phase describing the transfer function between the load pressure and nozzle pressure.

#### 8.1.2. Holding Phase

The holding phase is analysed in a similar manner as the fill phase. In the holding phase, the movement of the cylinder is negligible, as the only polymer flow into the mould is to counteract shrinkage. It is, therefore, possible to assume Δx≈0, which implies $V_{1}\left(x\right)\approx V_{1}$. The state vector is defined as Equation (43).

(43) $$\begin{array}{c} x_{Pack}={\left[\begin{array}{ccccccccccccc} x & \dot{x} & p_{1} & p_{2} & p_{3} & p_{4} & p_{5} & p_{6} & Q_{1} & Q_{2} & Q_{3} & Q_{4} & Q_{5} \end{array}\right]}^{T} \end{array}$$

The equilibrium point is calculated from $\ddot{x}=0$, ${\dot{p}}_{1}=0$, ${\dot{p}}_{2}=0$, ${\dot{p}}_{3}=0$, ${\dot{p}}_{4}=0$, ${\dot{p}}_{5}=0$, ${\dot{p}}_{6}=0$, ${\dot{Q}}_{1}=0$, ${\dot{Q}}_{2}=0$, ${\dot{Q}}_{3}=0$, ${\dot{Q}}_{4}=0$, ${\dot{Q}}_{5}=0$. The state space system is derived in a similar manner as the fill phase, as shown in Equation (38). This, however, requires an equation for each of the pressure and flows gradients. The leakage term that describes the shrinkage is pressure-dependent and is included as ${\dot{v}}_{i}\approx C_{i}\cdot p_{i}$. Due to the number of states the system matrix $A_{Pack}$ has, the size is 13×13, and it is omitted here. The transfer function is created similar to Equation (42). The linearisation is based on the equilibrium point of the system; this is possible due to the assumed constant volume of the barrel. Having described the linear systems for the both the fill and holding phases, the nozzle pressure controller can be designed.

### 8.2. Controller Design

The controller is formulated based on the transfer function of the fill phase (Equation (42)). All the requirements of closed-loop dynamics are not precisely defined. For the designed controller to be applicable to a range of injection moulding machines, it is desired to design the control system as a cascaded controller structure. The benefit of a cascaded controller structure is, if the outer loop is designed appropriately, the inner loop, in this case the injection moulding machine, is assumed not to impact the dynamics of the outer loop. The designed controller is, therefore, general for all injection moulding machines without the need to understand the machine dynamics in detail. The choice of a cascaded controller structure limits the desired closed-loop bandwidth to be a minimum of 10 times slower than that of the machine (60 rad/s). This machine is considered to be improved compared to a standard industrial injection moulding machine, meaning the bandwidth needs to be well below 6 rad/s.

**Figure 4:**
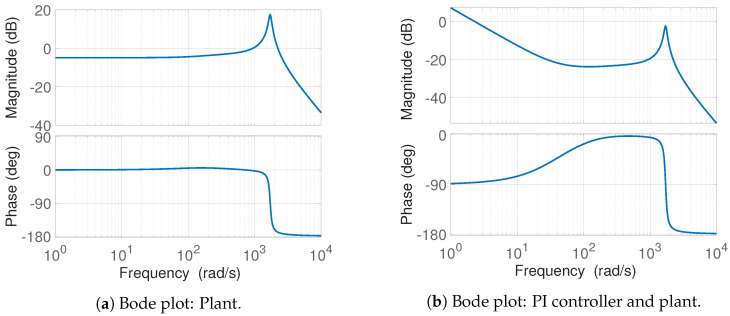
Open loop Bode plots for the (**a**) plant and the (**b**) controller and plant.

Repeatability is of great importance in injection moulding, and a steady flow of plastic increase the flow length as the shear heat reduces the cooling of the flow channel, meaning pressure fluctuations are undesired. This makes it desired to ensure the system is well damped without oscillations and zero steady state error for a step input. The open loop poles are all in the left half-plane, making it feasible to analyse the desired controller and gains from the open loop Bode plot shown in Figure 4a. The Bode plot is based on the linear model and is linearised at the point when the cavity is full, as this is found to be the instance with the lowest damping. From the figure, it can be seen that a significant resonant peak is present.

Multiple controller structures can yield the desired bandwidth; however, the desire for zero steady state error of a step input is one of the benefits of a PI controller. An additional benefit of a PI controller is that it damps high-frequency components, whereas, e.g., phase-lead controllers accentuate high-frequency noise. Further, PI controllers are well known in industry, meaning tuning, if necessary, can be conducted at the machine, as the functions of the two gains are well understood. The controller type chosen is a linear PI controller, with the controller structure and gains shown in Equation (44).

(44) $$\begin{array}{c} G_{C,1}=\frac{K_{p,1}s+K_{p,1}K_{i,1}}{s}\;,\;\;\;K_{i,1}=0.1\;,\;K_{p,1}=40 \end{array}$$

The Bode plot of the PI controller and plant is shown in Figure 4b. The gains are chosen to ensure a well-damped response, with a bandwidth well below the limit of 6 rad/s. The controller ensures a stable system, as all poles are in the left half-plane, and the controller results in a bandwidth of approximately 2.5 rad/s for the considered case. The design further results in an infinite gain and phase margin in the fill phase.

The stability of the controller is further assessed with respect to the linear model of the holding phase, which shows that all poles and zeros are in the left half-plane. The controller is deemed stable in the analysed area and is implemented for testing on the nonlinear model and the experimental setup.

## 9. Experimental Validation

The described model is verified and tested on an experimental setup consisting of a retrofitted industrial injection moulding machine. The original machine orchestrates the full process except for the injection phase, as the injection cylinder is driven by the novel electro–hydraulic variable-speed drive. The machine is equipped with a sensor in the nozzle, as shown in Figure 3, and two pressure sensors in the inlet and two pressure sensors in the cavity, as shown in Figure 2. All retrofitted equipment communicates through a commercial bus system with a frequency of 4 kHz. The material used for testing is an amorphous polymer called acrylonitrile butadiene styrene (ABS).

### 9.1. Validation: Nonlinear Model

A series of tests is performed, where the boundary conditions are varied. The process selected for comparison is the industry standard and is called secondary overpoint [4]. The two main stages are a velocity-controlled fill phase and a pressure-controlled holding phase. The test is performed as a full factorial design of experiments (DoE) with three factors in two levels. The factors considered are material mass temperature, injection velocity and holding pressure. The rest of the parameters for the moulding machine are kept constant. The DoE is performed in two blocks, with temperature as the blocking parameter. The presented data are for a representative cycle at steady-state production. Two of the eight test lines are presented, which are representative of the accuracy of the model. The tests selected are challenging, as temperature, injection velocity and holding pressure are all different for each test. The pressure sensors in the model are not positioned in the centre of each section, but instead, they are at both ends. This is true for both the runner and the cavity. The model estimates the pressure at the centre of each section. To compare the experiments with the model, the average of the two pressure sensors in the runner and cavity are used and are, respectively, denoted by ${\bar{•}}_{i}$.

**Figure 5:**
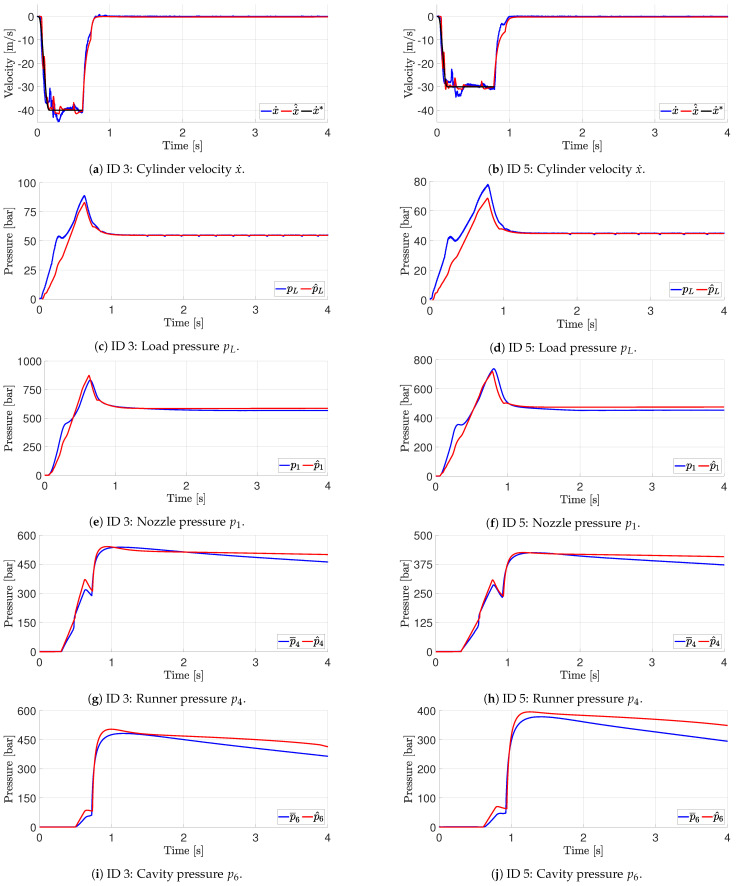
Simulation performance compared to experimental data of the described machine and mould: $\bar{•}$ is the average pressure; $\hat{•}$ is the simulation result; ${•}^{*}$ is the velocity reference.

The test parameters are shown in Table 2. The comparison of the experiments and model response are shown in Figure 5. The results are only plotted until approximately gate closure as this is the area of interest for controller design, as it is not possible to influence the pressure in the cavity after gate closure. The hydraulic load pressure is lowered to zero when the gate is closed.

The experimental results and the result from the proposed model are shown in Figure 5 for both ID 3 and ID 5. Figure 5a,b show the cylinder velocity reference together with the actual measured and modelled cylinder velocities. All regions are represented well in the model. Considering the load pressure shown in Figure 5c,d, the transient regions are well-modelled with an offset. The offset is also present in the nozzle pressure comparison shown in Figure 5e,f. The cavity pressure is plotted in Figure 5g,h and shows the same behaviour with good representation of the transient regions.

For all plots, the timings of some of the events, such as pressure changes and absolute values, show a difference; however, that is expected, as the model is greatly simplified and, e.g., is based on hydraulic diameters. From the plots, it is deemed that the model captures the dynamics of the moulding process in the tested region sufficiently to utilise the model for controller design.

### 9.2. Validation: Control Performance

**Figure 6:**
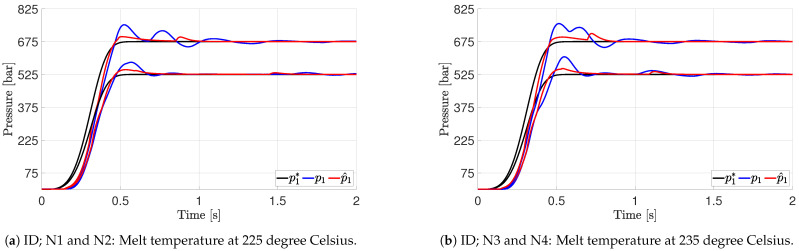
Performance of nozzle pressure controller for two set points and two temperatures evaluated on test machine and nonlinear simulation model. Reference pressure is denoted ${•}^{*}$, and simulation results are denoted with $\hat{•}$.

The controller designed in Section 8 is implemented in the model and the test system to test its performance using the nonlinear model and the experimental setup. A series of experiments is performed at two temperatures with two nozzle pressure references. The overview can be seen in Table 3. The simulation and test results are shown in Figure 6.

The results show good agreement between simulated and experimental data. A small overshoot is seen on both the model and test results, which is attributed to the hydraulic load pressure controller not being able to completely realise the desired inner-loop reference. The impact when the mould is volumetrically filled can be seen just before 1 second, as the nozzle pressure deviates from the reference shortly as the cylinder speed changes at a fast rate. The tests verify that the model is sufficient for control design and that the designed controller is stable and enables control of the nozzle pressure.

## 10. Discussion

This paper has presented a method for the development of a 1D nonlinear model that sufficiently describes the dynamics of an injection moulding machine and an injection moulding process for model-based controller design. A PI controller is designed and is proven effective for controlling the nozzle pressure both in the conducted experiment and in simulations.

The model relies heavily on mould geometry and material data. The timing of some of the events of the model, such as pressure changes and DC gain, could possibly be improved if the geometry is considered in greater detail. Additional material testing could possibly also improve the parameters as, e.g., the viscosity and Tait parameters are based on a single laboratory measurement. Tuning of both the material and geometric parameters will possibly increase the precision of the model; however, this will limit the use of the model to design controllers upfront before the setup is produced.

In general, the possibilities for model-based design of controllers are improved based on the developed model, as it is based solely on hydraulic diameters and material data. It is possible to consider system dynamics and design controllers without previous testing. As the model describes the pressure in each part of the machine and mould, it is possible to design controllers for these areas depending on the desired output; this is exemplified by the designed nozzle pressure controller.

The model considered in this paper describes a relatively simple mould geometry in a single-cavity tool. The model is sensitive to the selection of boundary conditions, as the boundary conditions have a great influence on the result. This can be a challenge for the presented method, and further investigation is needed to conclude how the model behaves when considering multi-cavity tools and shapes with complex geometric features.

If it is desired to further improve the nozzle pressure controller, the first assumption to evaluate is the assumption of an ideal load pressure machine controller. The inner loop is pressure-controlled, and unknown movement of the cylinder reduces the tracking capabilities of the inner loop. This could be addressed utilising, e.g., a velocity observer or working with a velocity reference instead.

## 11. Conclusions

A data-driven 1D nonlinear model is proposed that is especially developed for controller design based on mould geometry and material properties. The model describes the moulding process of an injection moulding machine retrofitted with an electro–hydraulic speed-variable actuator, producing in a single cavity tool with an amorphous ABS plastic. The model is verified experimentally at two temperatures, two injection speeds and two holding pressures. Based on a linearization of the nonlinear model, a model-based PI controller is designed with a desired bandwidth. The designed controller is tested both in experiments and on the developed model and it is proven that the desired tracking capabilities is obtained. The work presented enables the possibility for analysis of the described machine and mould dynamics and model-based control design. Further work is needed to generalise the described method, e.g., in moulds with additional complexity.

## Figures and Tables

**Table 1:** Geometric properties of the mould. For those of the sprue, see Section 6.

Parameter	Nozzle	Extender	Sprue	Runner	Gate	Cavity	Unit
*i*	1	2	3	4	5	6	-
$A_{c,i}$	70.68	25.51	-	26.29	4.5	40.39	mm^2^
$A_{s,i}$	-	2148	-	3948	26.69	4032	mm^2^
$D_{H,i}$	30	5.7	-	5.120	2	5.086	mm
$H_{T,i}$	-	120	87.3	192.2	2.966	127	mm
$V_{t,i}$	-	3062	2171	5054	13.34	5128	mm^3^

**Table 2:** Selected DoE test settings.

ID	Mass Temperature	Injection Velocity	Hydraulic Holding Pressure
	$\left({\bar{T}}_{I}\right)$ [^∘^C]	$\left(\dot{x}\right)$ [mm/s]	$\left(p_{L}^{*}\right)$ [bar]
3	225	40	55
5	235	30	45

**Table 3:** Settings for test of nozzle pressure controller.

ID	Mass Temperature	Nozzle Pressure Target
	$\left({\bar{T}}_{I}\right)$ [^∘^C]	$\left(p_{1}^{*}\right)$ [bar]
N1	225	525
N2	225	625
N3	235	525
N4	235	625

**Figure A1:**
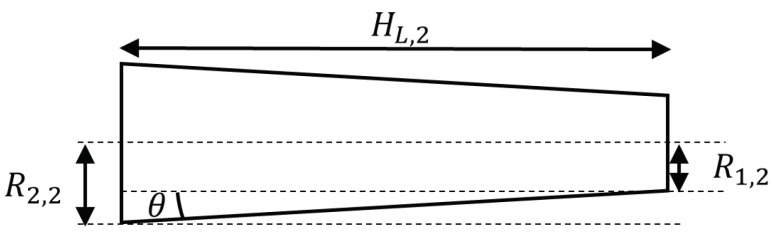
Geometry of the sprue.

## Data Availability

The datasets presented in this work are not available because of commercial considerations. Requests to access the data should be directed to the corresponding author.

## Notation

${•}^{*}$	Reference
${•}_{0}$	Linearisation point
${•}_{I}$	Initial condition
${•}_{i}$	*i* refers to a volume in the nozzle, extender, sprue, runner, gate or cavity and can take the value 1–6
${•}_{j}$	*j* refers to the sensor position in the given section *i* and can take the value 1–2
${•}_{k}$	Hydraulic chamber and can take the value A or B
$\ddot{•}$	Double time derivative
$\dot{•}$	Time derivative
$\bar{•}$	Average
$\tilde{•}$	Estimated value

## Nomenclature

$A_{c,i}$	Cross-sectional area of the i-th section
$A_{k}$	Area of cylinder chamber A
$A_{s,i}$	Surface area of the i-th section
$B_{v}$	Viscous friction
$D_{k}$	Displacement of pump k
$D_{H,i}$	Hydraulic diameter of the i-th section
$D_{\psi ,i}$	Hydraulic diameter after cooling of the i-th section
$F_{C}$	Coulomb friction
$F_{s,i}$	Resistive shear force of the i-th section
$G_{C,i}$	Controller for section i
$H_{L,i}$	Hydraulic length of the i-th section
$K_{i,i}$	Integral gain of controller i
$K_{p,i}$	Proportional gain of controller i
$Q_{i}$	Flow of the i-th section
$Q_{k}$	Flow from pump k
$Q_{Lc}$	Leakage flow across cylinder
$T_{g}$	Glass transition temperature
$T_{s}$	Cooling water temperature
$T_{W}$	Temperature of the mould
$T_{W}$	Peak temperature of the polymer
$T_{i}$	Temperature of the i-th section
$V_{i}$	Volume of the i-th section
$V_{k}$	Volume of hydraulic chamber k
$\beta_{A}$	Bulk modulus of hydraulic chamber k
$\beta_{i}$	Bulk modulus of the i-th section
${\dot{\gamma }}_{i}$	Shear rate of the i-th section
$\eta_{0}$	Zero shear viscosity
$\eta_{i}$	Viscosity of the i-th section
${\hat{p}}_{i}$	Pressure in the i-th section
$\kappa_{i}$	Adiabatic compressibility of the i-th section
$\nu_{i}$	Specific volume of the i-th section
$\nu_{p}$	Poisson ratio
$\omega_{H}$	Bandwidth of level pressure controller
$\omega_{L}$	Bandwidth of load pressure controller
$\psi_{i}$	Percentage of frozen layer
$\rho_{i}$	Density of the i-th section
$\tau_{rz,i}$	Shear stress of the i-th section
*b*	Two-domain Tait parameters
$b_{p}$	Thermal effusivity of the polymer
$b_{s}$	Thermal effusivity of the mould
$c_{p}$	Specific heat of the plastic
*k*	Thermal conductivity of the polymer
*m*	Mass of injection unit and screw
$m_{i}$	Mass of the i-th section
$p_{H}$	Hydraulic level pressure
$p_{L}$	Hydraulic load pressure
$p_{k}$	Hydraulic pressure in chamber k
$p_{hold}$	Holding pressure
*t*	Time
$x_{dec}$	Decompression distance
$x_{s}$	Starting point of ram

## Appendix A. Hydraulic Dimensions: Sprue

For the sprue, as it is conical, it is not possible to only scale the flow path; it is further not possible to utilise a single area. The sprue is shown in Figure A1.

The radius $R_{2,2}$ is dependent on the amount of plastic in the sprue and is calculated from the volume of material in the sprue, as shown in Equation (A1).

(A1) $$\begin{array}{c} R_{2,2}={\left(\frac{3\cdot V_{2}\tan\left(\theta \right)}{\pi }+R_{1,2}^{3}\right)}^{\frac{1}{3}} \end{array}$$

The logarithmic mean between $R_{1,2}$ and $R_{2,2}$ is used to calculate the hydraulic diameter of the sprue, as given in Equation (A2)

(A2) $$\begin{array}{c} D_{H,3}=2\frac{R_{2,2}-R_{1,2}}{log\left(\frac{R_{2,2}}{R_{1,2}}\right)} \end{array}$$

For the case of $log\left(\frac{R_{2,2}}{R_{1,2}}\right)\approx 0$, the logarithmic mean radius is $r_{hyd,Lmean}=R_{1,2}$ to avoid dividing by zero. The hydraulic length $H_{L,i}$ is given by Equation (A3):

(A3) $$\begin{array}{c} H_{L,3}=\frac{R_{2,2}-R_{1,2}}{\tan(\theta )} \end{array}$$

The cross-sectional area can then be calculated as Equation (A4):

(A4) $$\begin{array}{c} A_{c,3}=\frac{D_{H,3}^{2}\cdot \pi }{4} \end{array}$$

The surface area of the sprue $A_{s,2}$ can be calculated as the lateral surface area of a truncated cone, as in Equation (A5).

(A5) $$\begin{array}{c} A_{s,3}=\pi \left(R_{2,2}+R_{1,2}\right)H_{L,3} \end{array}$$
